# Trio exome analysis is a valuable tool for genetic diagnosis of epilepsy in Mali

**DOI:** 10.1016/j.gimo.2025.103449

**Published:** 2025-08-13

**Authors:** Salia Bamba, Lauren Jeffries, Salimata Diarra, Karamoko Nimaga, Amadou Touré, Modibo K. Goita, Seybou H. Diallo, Weizhen Ji, Alassane Baneye Maiga, Oumou Traoré, Moussa Doumbia, Adama Koné, Koudoussou Olaréwadjou Sanni, Ahmed Camara, Mohamed A. Cissé, Ramatoulaye Kane, Ibrahim Nimaga, Mamadou Traoré, Lassana Cissé, Abdoulaye Yalcouyé, Cheick Abdel kader Cissé, Samuel Ephrata Mefoung, Moussa Sangaré, Mahamadou Kotioumbé, Aissata S. Touré, Mohamed Emile Dembélé, Emily K. Mis, Cheick Oumar Guinto, Oumar Samassékou, Mahamadou Traoré, Mustafa K. Khokha, Guida Landouré, Saquib A. Lakhani

**Affiliations:** 1 Faculté de Médecine et d’Odontostomatologie, Université des Sciences, des Techniques et des Technologies de Bamako, Bamako, Mali; 2 Department of Pediatrics, Cedars Sinai Guerin Children’s, Los Angeles, CA; 3 Pediatric Genomics Discovery Program (PGDP), Department of Pediatrics, Yale University School of Medicine, New Haven, CT; 4 Clinique Dinandougou, Markacoungo, Mali; 5 Service de Pédiatrie, Centre Hospitalier Universitaire de Gabriel Touré, Bamako, Mali; 6 Clinique Kaïdara, Bamako, Mali; 7 Service de Neurologie, Centre Hospitalier Universitaire de Gabriel Touré, Bamako, Mali; 8 Hôpital Nianankoro Fomba de Ségou, Ségou, Mali; 9 Service de Neurologie, Centre Hospitalier Universitaire du Point G, Bamako, Mali

**Keywords:** Africa, Epilepsy, Exome sequencing, Mali, Trio analysis

## Abstract

**Purpose:**

Trio exome sequencing is widely used for various disorders. We investigated the utility of this method to identify genetic causes of epilepsy in Mali.

**Methods:**

We enrolled patients with epilepsy suspected of an underlying genetic etiology. Exome sequencing data from those with a minimum of a trio (proband and both biological parents) were analyzed to identify and classify potential causative genetic variants.

**Results:**

We sequenced 159 individuals, including 57 patients with epilepsy from 42 families, the largest trio sequencing cohort for epilepsy in sub-Saharan Africa. Of these, 16 families (38%) received a putative molecular diagnosis, with autosomal recessive inheritance seen in 7 of 16 (44%) and de novo events in 9 of 16 (56%). The 17 total variants were classified as pathogenic (*n* = 8) or likely pathogenic (*n* = 9), with 14 of 17 (82%) being novel to public databases. An additional 6 of 42 families (14%) had variants of uncertain significance with consistent genotype-phenotype correlations. There were no common candidate genes across families.

**Conclusion:**

Our findings illuminate a significant genetic contribution to epilepsy in Mali with a substantial genetic heterogeneity, as well as the utility of trio exome sequencing for efficient diagnosis. This further emphasizes the persistent and critical need for greater inclusivity in genomic research and implementation.

## Introduction

Epilepsy is a global health challenge, affecting around 50 million people worldwide, including an estimated 25 million in Africa.^1^ The high prevalence in Africa is thought to be driven by environmental factors such as infections, perinatal complications, traumatic brain injuries, and limited access to health care resources.^2^ However, the contribution of genetic factors to epilepsy in Africa, particularly in sub-Saharan Africa, remains largely unexplored.^3^

Advances in next-generation sequencing (NGS) have transformed the diagnostic approach to epilepsy, enabling a greater number of genetic diagnoses.^4^ A recent meta-analysis found diagnostic rates in epilepsy patients of 24% (95% CI 18%-30%) for exome sequencing (ES) and 48% (95% CI 28%-70%) for genome sequencing.^5^ Trio sequencing, involving both biological parents in addition to the proband, has been shown to be more efficient, not only reducing false positives but also enhancing the prioritization of potential disease-causing variants, with a 5%-10% increase in diagnostic yield compared with analyzing the proband alone.^6^

However, most epilepsy genetic and genomic studies have focused on people with European ancestry. In contrast, genetic epilepsies are among the most under-investigated neurological disorders in Africa, and little knowledge currently exists on the genetics of epilepsy across the continent.^7^^,^^8^ Given the limited access to genetic testing in Africa, genetic epilepsies and associated syndromes are frequently overlooked or misdiagnosed, leading to suboptimal and even inappropriate treatment approaches.^9^

In this study, we sought to better define the genetic contribution to epilepsy in Mali, a resource-limited landlocked country in sub-Saharan Africa. To optimize the use of resources, we first screened patients to exclude those with suspected nongenetic causes of epilepsy, then utilized ES with trios (proband and both biological parents) to maximize the chances of identifying potential diagnostic genes. Our results demonstrate a significant burden of underlying genetic etiologies for epilepsy in Mali, findings with important implications for diagnostic and management approaches.

## Materials and Methods

### Study population

We recruited patients with a clinical diagnosis of epilepsy from a tertiary care center in Mali. Neurologists conducted comprehensive evaluations, collecting data on demographics (including self-reported ethnicity and geographic region of origin), seizure type and management, disease progression, family history and pedigree, clinical examination findings, and results from ancillary studies including laboratory tests, electroencephalography (EEG), and brain magnetic resonance imaging. Exclusion criteria for this study were (1) history or examination findings indicative of a nongenetic seizure etiology, such as perinatal events, brain infections, head trauma, or stroke and (2) the unavailability of 1 or both biological parents, which were required for trio ES.

### Exome sequencing and analysis

Genomic DNA was extracted from peripheral blood using DNA Puregene Blood Kit (QIAGEN). NGS was performed at a Genome Analysis. Exome capture was performed using xGen target capture kit from IDT followed by 99 base paired-end sequencing on the Illumina platform with target read depths of 80× for probands and 40× for parents and additional relatives. More than 95% of the targeted region was covered over 20×.

Reads were aligned to the human genome reference assembly (UCSC Genome Browser hg19) with the Burrows-Wheeler Aligner. The Genome Analysis Toolkit version 4.0 (GATK4.0, Broad Institute) was used to remove polymerase chain reaction duplicates and evaluate the quality of variants by attaining effective reads, effective base, average coverage depth, and coverage ratio. Single-nucleotide variants and short insertions and deletions (indels) were also called by GATK4. The sex of all individuals undergoing ES were verified using plinkv1.9. Familial relationships were verified via Identity-by-Descent sharing (plinkv1.9) and the Kinship-based Inference for GWAS (KING) algorithm.

ANNOVAR (version 20191024) and VEP (version 111) were used to functionally annotate variants with a multiple pathogenicity prediction score including evaluations from Sorting Intolerant From Tolerant (SIFT), SIFT-4G, PolyPhen-2 (HDIV and HVAR), MutationTaster, Mendelian Clinically Applicable Pathogenicity (M-CAP), MetaSVM, MetaLR, FATHMM, LRT, MutationAssessor, PROVEAN, MVP, MPC, VEST4, REVEL, CADD, regSNP-intron, Spidex, and dbscSNV11. Conservation and intolerance scores GERP++, PhyloP (46-way, 100-vertebrate, and 30-mammalian), pLI, and CADD Conservation, were also included. Population frequency data were obtained from the 1000 Genomes Project and the Genome Aggregation Database (initially gnomAD V2.1.1 with confirmation using gnomAD 4.1.0). Additionally, variants were annotated using ClinVar (Evaluation and Phenotype), Online Mendelian Inheritance in Man, Human Phenotype Ontology, GenCC Disease, Gene Ontology (GO) Function, GO Cell Component, GO Pathway, Kyoto Encyclopedia of Genes and Genomes Pathway, Reactome, and InterPro. R programming language was used to prioritize candidate variants based upon segregation, annotation, and allelic frequencies of less than <0.05%, as well as interrogation of known epilepsy genes.^10^ Candidate variants were visualized and verified manually by Integrative Genomics Viewer (https://igv.org/) and classified according to American College of Medical Genetics and Genomics (ACMG) criteria using VarSome (https://varsome.com/) with manual verification using ClinGen guidelines.^11^, ^12^, ^13^

## Results

We observed 154 families with at least 1 individual referred for suspected epilepsy and 136 (88%) provided consent to participate; 3 families (2%) declined to consent, and 1 family (1%) deferred a decision on consent and then was lost to follow up. Additionally, 14 families (9%) were excluded during screening due to a suspected nongenetic etiology for seizures.

Following evaluation, at least a trio was available in 42 of the 136 consenting families (31%), comprising 15 trios, 24 quartets and 3 sextets. Supplemental Table 1 provides clinical and sociodemographic data for these 42 families. Two families originated from Burkina Faso, 1 each from the ethnic groups Dafing and Mossi. The remaining families represented 9 of the 13 major Malian ethnic groups: Bambara 16/42 (38%), Soninke 9/42 (21%), Fulani 8/42 (19%), Malinke 2/42 (5%), and 1/42 (2%) each from Dogon, Kakolo, Kassoke, Senoufo, and Songhai. Geographically, the families represented 7 of 10 regions in Mali: Koulikoro 18 of 42 (42%), Ségou 9 of 42 (21%), Kayes 8 of 42 (19%), Mopti 2 of 42 (5%), Sikasso 2 of 42 (5%), and 1 of 42 (2%) each from Gao and Tombouctou, as well as 1 of 42 (2%) from the capital district of Bamako. Consanguinity was reported in 19 of 42 (45%) of these families and 28 of 42 (67%) of the families had more than 1 affected individual with seizures. Patients with epilepsy evaluated for this study were predominantly children under the age of 18 years (45/57 = 79%) and all except 1 had onset of seizures before 18 years of age. In addition to seizures, some patients displayed a spectrum of additional clinical features, such as developmental delay and hypotonia.

**Figure 1:**
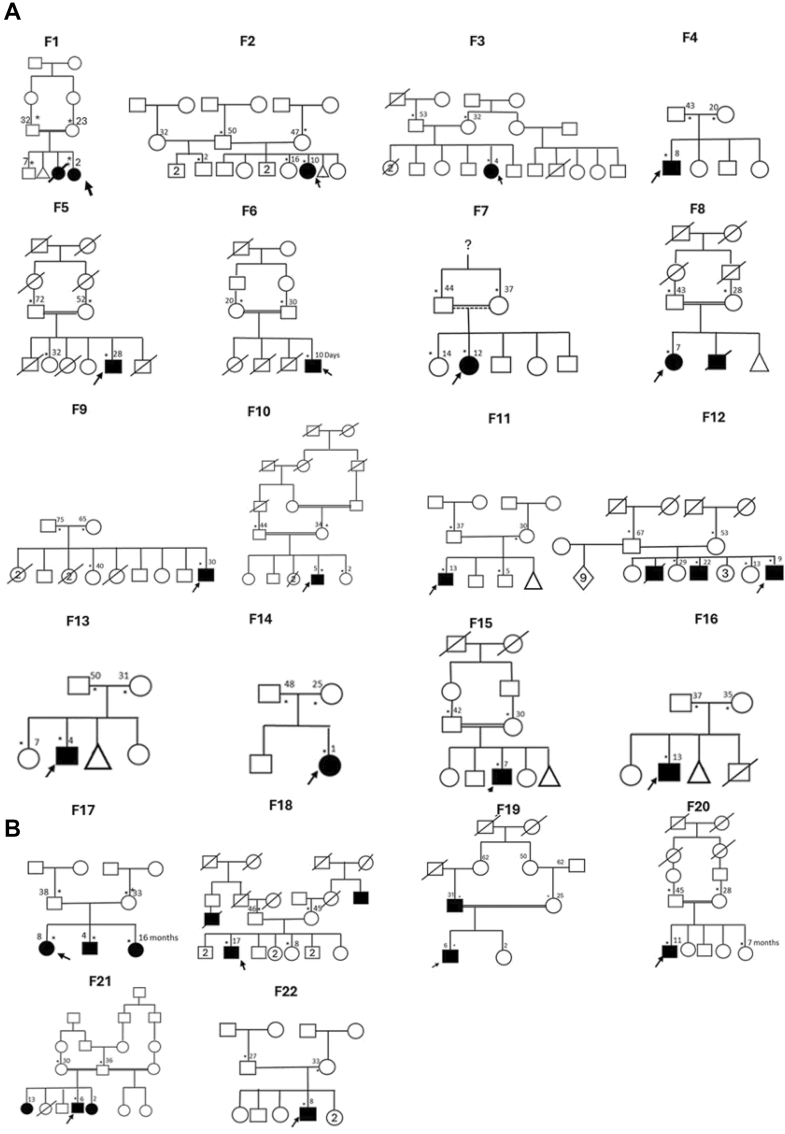
**Pedigrees of 22 families with genes variants of interest consistent with epilepsy phenotypes.** A. Pedigrees of the 16 Malian families with diagnostic pathogenic or likely pathogenic gene variants, by ACMG criteria. B. Pedigrees of the 6 Malian families with variant of uncertain significance in genes with clinical phenotypes consistent with seizures. For all pedigrees, probands are indicated with an arrow. Affected individuals with seizures are shaded black. Small asterisk (∗) next to individual symbols indicates that the person was evaluated in clinic and small number indicates age at evaluation.

From these 42 families, we sequenced 57 patients with epilepsy and 102 healthy family members, for a total of 159 individuals. Exome analysis found strong candidate variants in genes consistent with the clinical phenotype in 16 of 42 (38%) of the families (Figure 1A, Table 1). One family had a compound heterozygous variant with the remaining families all having single variants, resulting in a total of 17 distinct diagnostic variants. Using ACMG criteria with ClinGen guidelines, 8 of 17 (47%) variants were classified as pathogenic and 9 of 17 (53%) as likely pathogenic. Notably, each family had candidate variants in a unique gene, with no overlap across families. Missense was the most prevalent candidate variant type, comprising 8 of 17 (47%), followed by nonsense 5 of 17 (29%), splice site variants 3 of 17(18%), and a single frameshift deletion 1 of 16 (6%). Autosomal recessive inheritance was observed in 7 of 16 families (44%), and de novo variants were detected in 9 of 16 families (56%). Overall, 14 of 17 (82%) of the variants had not been previously reported in public databases. Additionally, we identified 6 families with variants of uncertain significance (VUS) in genes having defined clinical presentations matching the phenotype associated with the gene of interest (Figure 1B, Supplemental Table 2).

**Table 1:** Molecular diagnosis and presumptive diagnostic genes for patients with ACMG criteria

Family	Consanguinity (Yes/No)	Gene (HGNC ID)	Gene Name	Genomic Variant	DNA and Protein Variant		ACMG Criteria	Inheritance Pattern	Novel Variant (Yes/No)
F1	Yes	*HEXA* (HGNC:4878)	Hexosaminidase subunit alpha	NC_000015.9:g.72648896G>A	NM_000520.6: c.[316C>T];[316C>T]	NP_000511.2: p.(Gln106Ter)	P (PVS1 PM2 PM3)	AR	No
F2	No	*NOTCH3* (HGNC:7883)	Notch receptor 3	NC_000019.9:g.15281611T>G	NM_000435.3: c.4762A>C	NP_000426.2: p.(Asn1588His)	LP (PS2 PM2 PP2 PP3 PP4)	De novo	Yes
F3	No	*ARFGEF1* (HGNC:15772)	ADP ribosylation factor guanine nucleotide exchange factor 1	NC_000008.10:g.68179441T>G	NM_006421.5: c.1697A>C	NP_006412.2: p.(Tyr566Ser)	LP (PP3 PM2 PS2 BP1 PP4)	De novo	Yes
F4	No	*DEPDC5* (HGNC:18423)	DEP domain containing 5	NC_000022.10:g.32217496G>C	NM_001242896.3: c.1879G>C	NP_001229825.1: p.(Gly627Arg)	LP (PS2 PM2 PP4)	De novo	Yes
F5	No	*LARP7* (HGNC:24912)	La ribonucleoprotein domain family member 7	NC_000004.11:g.113578402G>A	NM_001370978.1: c.[1669-1G>A];[1669-1G>A]	NP_001357907.1: p.?	P (PVS1, PM2, PM3)	AR	Yes
F6	Yes	*ATIC* (HGNC:794)	5-aminoimidazole-4-carboxamideribonucleotide formyltransferase/IMP cyclohydrolase	NC_000002.11:g.216177245C>A	NM_004044.7: c.[44C>A];[44C>A]	NP_004035.2: p.(Thr15Asn)	LP (PM2 PM3 PP4_strong)	AR	Yes
F7	Yes	*WFS1* (HGNC:12762)	Wolframin ER transmembrane glycoprotein	NC_000004.11:g.6303561A>C	NM_006005.3: c.[2039A>C];[2039A>C]	NP_005996.2: p.(Glu680Ala)	LP (PM1 PM6 PM3 PP4)	AR	No
F8	Yes	*ST3GAL3* (HGNC:10866)	ST3 beta-galactoside alpha-2,3-sialyltransferase 3	NC_000001.10:g.44365212G>A	NM_174963.5: c.[765-1G>A];[765-1G>A]	NP_777623.2: p.?	P (PVS1 PM2 PM3 PP4)	AR	Yes
F9	No	*SCN8A* (HGNC:10596)	Sodium voltage-gated channel alpha subunit 8	NC_000012.11:g.52078043T>A	NM_014191.4: c.362T>A	NP_055006.1: p.(Ile121Lys)	LP (PS2 PM2 PP3 PP4)	De novo	Yes
F10	Yes	*GRIA1* (HGNC:4571)	Glutamate ionotropic receptor AMPA type subunit 1	NC_000005.9:g.153077655C>T	NM_000827.4: c.1186C>T	NP_000818.2: p.(Gln396Ter)	P (PS2 PP3 PM2 PP4)	De novo	Yes
F11	No	*CHD2* (HGNC:1917)	Chromodomain helicase DNA binding protein 2	NC_000015.9:g.93499880G>T	NM_001271.4: c.2000+1G>T	NP_001262.3: p.?	P (PVS1 PM2 PS2 PP4)	De novo	Yes
F12	Yes	*AARS1* (HGNC:20)	Alanyl-tRNA synthetase 1	NC_000016.9:g.70287873_70287875del; NC_000016.9:g.70292033C>T	NM_001605.3: c.[2470_2472del; 2080G>A]	NP_001596.2: p.(Lys824del) NP_001596.2: p.(Val694Met)	LP (PP1 PP4 PM2 PM4) LP (PP1 PP4 PM3 PM2 PP3)	AR	Yes
F13	No	*TSC2* (HGNC:12363)	Tuberous sclerosis complex 2	NC_000016.9:g.2105519C>T	NM_000548.5: c.598C>T	NP_000539.2: p.(Gln200Ter)	P (PVS1 PM2 PS2 PP4)	De novo	Yes
F14	No	*TSC2* (HGNC:12363)	Tuberous sclerosis complex 2	NC_000016.9:g.2134598C>T	NM_000548.5: c.4375C>T	NP_000539.2: p.(Arg1459Ter)	P (PVS1 PS2 PP4)	De novo	Yes
F15	Yes	*TPP1* (HGNC:2073)	Tripeptidyl peptidase 1	NC_000011.9:g.6638271G>A	NM_000391.4: c[.622C>T];[622C>T]	NP_000382.3: p.(Arg208Ter)	P (PVS1 PM2 PP4)	AR	No
F16	No	*KANSL1* (HGNC:24565)	KAT8 regulatory NSL complex subunit 1	NC_000017.10:g.44143947G>C	NM_015443.4: c.1804C>G	NP_056258.1: p.(Arg602Gly)	LP (PS2 PM2 PP4)	De novo	Yes

*AR*, autosomal recessive, HGNC, HUGO Gene Nomenclature Committee; *LP*, likely pathogenic; *P*, pathogenic.

## Discussion

This study presents a large cohort of trio ES for epilepsy diagnosis in sub-Saharan Africa, offering a broad overview of the genetic basis of this condition in Mali and emphasizing the importance of trio analysis. By screening out families with suspected nongenetic etiologies for their seizures, which serves as a practical means to aid implementation by maximizing yield in areas with limited resources, we were able to achieve a diagnostic rate of roughly 40%. This suggests that providing NGS in this population could greatly affect multiple facets of epilepsy diagnosis and management. Although practical limitations in accessing targeted antiseizure medications remains an ongoing issue in Africa even for actionable genes, genetic diagnoses did lead to invaluable enhancements in guidance, education, and family planning for the families that we evaluated.

The high rate of families with autosomal recessive variants is consistent with the observed consanguinity in our overall cohort (45%), with all but 1 of these being homozygous. Nevertheless, more than half of the families had de novo diagnostic variants, and all of these de novo variants were novel, with no prior reports in the literature, which is likely reflective of the limited prior sequencing of individuals from sub-Saharan Africa in known databases. This suggests that proband-only sequencing may be insufficient for epilepsy diagnostics in these populations, because de novo variants would need to be first flagged in a pipeline and subsequent targeted sequencing of parents would be needed to confirm de novo status. Furthermore, sequencing probands alone would not only reduce the possibility of identifying novel variants in known genes but would also diminish the chances of discovering de novo variants in novel seizure genes, which is a distinct possibility in populations that have not been well characterized by sequencing. In particular, the traditionally large families and high consanguinity in African family settings offer a unique opportunity to uncover previously unknown variants and genes.

It is notable that none of the 16 diagnosed families had overlapping diagnostic genes. This may have been influenced by our conscious attempt to recruit families from diverse ethnicities and locales that would be reflective of the entire country. Indeed, we were able to have participants that reflected all the regions of the country and from 9 ethnic groups, including the 3 most of common that make up more than half of the Malian population (Bambara 33% of general population, Fulani 13%, and Soninke 10%).^14^ Also, many of the genes and variants identified in our study have not been previously reported in African populations, adding to the genetic diversity observed in epilepsy within the Malian population. This also affects the fact that an additional 6 families had variants in genes with known phenotypes consistent with seizures that did not meet the threshold for pathogenicity and were thus classified as VUS. Additional sequencing efforts in underrepresented populations, along with appropriate functional studies, will aid in determining whether such VUS are benign or pathogenic. Regardless, this striking lack of genetic overlap speaks to the genetic heterogeneity of epilepsy in Mali. This diversity also suggests that broad exome or genome sequencing may be a better choice as opposed to panel-based testing, although larger numbers are needed to make this case definitively.

In summary, our findings illuminate a significant genetic contribution to epilepsy in Mali with a substantial genetic heterogeneity. We also demonstrate the value of trio ES in achieving accurate diagnoses for genetic epilepsies. Finally, our findings emphasize the persistent and critical need for greater inclusivity in genomic research, especially understudied populations such as sub-Saharan Africa.

## Data Availability

Data are available upon request from the authors.

## Declaration of AI and AI-Assisted Technologies in the Writing Process

Neither AI nor AI-assisted technologies were used in the writing process of this manuscript.

## Conflict of Interest

SAL and MKK are part owners of Victory Genomics, a startup company unrelated to this work. All other authors declare no conflicts of interest.
