# Supplementary material for: Trio exome analysis is a valuable tool for genetic diagnosis of epilepsy in Mali
Source: Genet Med Open. 2025 Aug 13;3:103449. doi: 10.1016/j.gimo.2025.103449 (PMC12481924; doi:10.1016/j.gimo.2025.103449)
Supplement: Supplementary Table 2 [file mmc2.pdf]

Additional Information

Supplementary Table I provides additional clinical and sociodemographic data for all 42 families evaluated for this study. Supplementary Table II provides additional variant information for families where candidate variants were classified as VUS but the patient phenotype was consistent with the known phenotype associated with the gene of interest.

Supplementary Table II: Candidate genes for families with variants classified as VUS by ACMG criteria where the patient seizure phenotype was consistent with the known phenotype associated with the gene

Family	Consanguinity	Gene	Gene name	DNA Variant	Protein Variant	ACMG Criteria + ClinGen	Inheritance Pattern	Novel Variant in gnomAD 4.1.0
F17	Yes	*HECW2*	HECT, C2, and WW Domain Containing E3 Ubiquitin Protein Ligase 2	NM_001348768.2: c.1856C>G	NP_001335697.1: p.(Pro619Arg)	VUS (PP1 PP2 PP4)	AR	No
F18	No	*NEXMIF*	Neuronal Expressed, Developmentally Downregulated X-Linked, Molecule Interacting with Forkhead Box M1	NM_015158.5: c.3400G>A	NP_055973.2: p.(Ala1134Thr)	VUS (PM2 PP1 PP4)	XLR	Yes
F19	No	*KANK1*	KN Motif and Ankyrin Repeat Domains 1	NM_006059.4: c.544G>A	NP_006050.3: p.(Gly182Ser)	VUS (PM2 PP4 BP1)	AD	Yes
F20	Yes	*ZMYM3*	Zinc Finger MYM-Type Containing 3	NM_024531.5: c.151G>A & NM_024531.5: c.155C>T	NP_078807.1 : p.(Val51Ile) & NP_078807.1 : p.(Ser52Phe)	VUS (PM2 PM3 BP4) & LP (PM2 PM5)	XLR	Yes
F21	No	*LAMC3*	Laminin Subunit Gamma 3	NM_015158.5: c.3400G>A	NP_055973.2: p.(Ala1134Thr)	VUS (PM2 PP1 PP4)	AR	No
F22	Yes	*SLC52A2*	Solute Carrier Family 52 Member 2	NM_024531.5: c.151G>A & NM_024531.5: c.155C>T	NP_078807.1 : p.(Val51Ile) & NP_078807.1 : p.(Ser52Phe)	VUS (PM2 PM3 BP4) & LP (PM2 PM5)	AR	No

Abbreviations: VUS= variant of uncertain significance, AR=Autosomal recessive, AD = Autosomal dominant, XLR= X-linked Recessive
